# Comparative study on eating habits and health of single-person and multi-person households

**DOI:** 10.1371/journal.pone.0327763

**Published:** 2025-07-07

**Authors:** Haerang Lee, Seon-Jip Kim, Minji Kang

**Affiliations:** 1 Research Institute of Human Ecology, Seoul National University, Seoul, Republic of Korea; 2 Department of Preventive Dentistry and Public Oral Health, School of Dentistry & Dental Research Institute, Seoul National University, Seoul, Republic of Korea; 3 Department of Food and Nutrition, Duksung Women’s University, Seoul, Republic of Korea; Keimyung University, KOREA, REPUBLIC OF

## Abstract

This study investigates the differences in health, dietary habits, and quality of life between single-person and multi-person households, with a specific focus on demographic subgroups such as gender and age. Data were drawn from the 2013–2021 Korean National Health and Nutrition Examination Survey, encompassing 40,839 participants aged 19 and older, and were analyzed using multivariate regression models. The results revealed significant disparities between the two types of households. Single-person male households exhibited poorer dietary quality and higher metabolic risk indicators, such as elevated waist circumference, low-density lipoprotein cholesterol, and blood pressure, compared to their multi-person counterparts. In contrast, single-person female households demonstrated slightly better metabolic profiles, including lower body mass index, triglycerides, and fasting glucose, relative to multi-person households. Quality of life, assessed using the EuroQol-5 Dimension, was significantly lower in single-person households, with women reporting higher rates of anxiety, depression, and mobility issues. Subgroup analyses identified pronounced vulnerabilities among single-person males aged 40–59 and single-person females aged 60 and older. These findings highlight the need for tailored public health policies and market strategies to address the distinct needs of diverse single-person household subgroups. Further research should prioritize well-designed studies exploring populations with unique characteristics, such as single-person households, to better understand their specific challenges and requirements compared to multi-person households.

## Introduction

Globally, single-person households have continued to rise [1]. A single-person household is defined as a household where an individual lives alone [2]. According to the OECD, in 2015, the proportion of single-person households averaged 30.7% across all households [3]. As of 2019, the proportion of single-person households was 44.7% in Finland, 42.3% in Germany, 29.5% in the United Kingdom, and 28.4% in the United States [1]. In South Korea, the proportion of single-person households was 34.5% of the total households in 2022, making it the largest type of household [4]. The rise in single-person households can be attributed to factors such as an increase in non-marriage and delayed marriage among younger generations, shifts in family values leading to higher divorce and separation rates, and population aging [5,6]. While single-person households were primarily composed of elderly individuals in the past, they have recently become more common across all age groups. When examining the composition of single-person households by age in various countries, 19.2% of single-person households in South Korea (Korea hereafter) are under the age of 29, the largest proportion, followed by 30–39-year-olds and 60–69-year-olds, who account for 16.7% [4]. In the United States, in 2020, 11.1% of individuals in single-person households were aged 15–64, while 16.5% were 65 and older [7].

As the diversity within single-person households has grown, the quality of life—including nutritional intake and mental health—can vary depending on demographic characteristics such as age, marital status, and gender. For instance, active seniors tend to have purchasing power and engage in various activities to enhance their quality of life. In contrast, single-person households with relatively lower purchasing power or those with limited income or physical abilities may be more vulnerable in terms of quality of life [8]. Studies have also indicated that females in single-person households often have better health outcomes, dietary quality, and lower disease prevalence compared to their male counterparts [9–11]. These differences have led to a growing consensus that research on single-person households should not simply compare them to multi-person households but should also consider the internal diversity of single-person households [12]. Differences in health and dietary patterns of single-person households are particularly evident. Thus, it is crucial to analyze single-person households according to their distinct characteristics.

Single-person households exhibit a higher prevalence of chronic and mental health conditions compared to multi-person households, which is associated with lower physical and mental health-related quality of life [13,14]. In terms of diet, studies have highlighted the vulnerability of single-person households, often showing less healthy dietary habits and health [15–17]. For example, single-person households are more likely to skip meals, have irregular eating patterns, and consume nutritionally imbalanced meals [16,18]. Their dietary quality and prevalence of chronic conditions are also lower compared to those in multi-person households. In particular, elderly individuals living alone face greater challenges in maintaining healthy eating habits and overall health compared to those living with family members [10]. These individuals also often lack familial support or robust social systems, making it challenging to maintain daily living activities and safely, which contributes to poorer physical health, especially chronic illnesses [19,20]. Furthermore, studies have reported that single-person female households have lower dietary satisfaction and quality and a higher incidence of illness compared to those living in multi-person households [5,6].

From a mental health perspective, single-person households have been found to experience mental health disorders more frequently than those in multi-person households [21,22]. Kim et al. (2017) found that single-person households in their 30s and 40s have a higher risk of suicide compared to multi-person households, while those in their 50s and 60s are more prone to stress and depressive symptoms.

With the rise in single-person households, more research has been conducted on their health, quality of life, and dietary habits. However, most studies have focused on specific age groups or genders, such as senior, middle-aged, or female single-person households [6,11,22]. However, given the growing diversity within single-person households, it is crucial to consider the diverse demographic characteristics. This study aims to compare the dietary habits and quality of life of single-person and multi-person households by disaggregating them based on demographic characteristics such as gender and age. This approach provides a more detailed understanding of the diverse aspects of quality of life and dietary patterns among single-person households. It also provides foundational information for developing targeted policies and market strategies for diverse single-person households.

## Method

### Study participants

This study utilized data from the 2013–2021 Korean National Health and Nutrition Examination Survey (KNHANES), a nationally representative, cross-sectional survey conducted by the Korea Disease Control and Prevention Agency (KDCA). The KNHANES uses a stratified, multistage probability sampling method that comprises three surveys: a health interview, a health examination, and a nutrition survey. Briefly, health interviews and health examinations were performed by trained medical staff and interviewers at a mobile examination center. The nutrition survey was completed by trained dietitians at the participants’ homes and included a 24-hour dietary recall, as well as a questionnaire on dietary behaviors. Further detailed information on the KNHANES is published elsewhere [23].

The study protocol of the KNHANES received approval from the KDCA Institutional Review Board (IRB approval number: IRB No. 2001-114-1096). The analysis included 40,839 participants (men = 17,234; women = 23,605) aged 19 years or older who completed the nutrition survey and did not report a history of major cardiovascular disease (stroke, myocardial infarction, or angina) or majors (stomach, liver, colorectal, breast, lung, or thyroid cancer).

### Household types

Participants were classified based on their responses to the question, “What type of household do you belong to?” Participants who selected “single-person household” were categorized as living in single-person households. Those who indicated living with a spouse, cohabitant, or as part of a two-generation or three-generation household were categorized as residing in multi-person households.

### Assessment of health-related factors

#### Dietary habits.

Dietary data were collected using a 24-hour dietary recall method, conducted by trained interviewers who conducted in-person visits to participants’ households. During the interviews, detailed information was obtained about all foods and beverages consumed in the past 24 hours, including the types of food consumed, eating location, and time of consumption. To improve the accuracy of portion size estimates, standardized aids such as food models, portion size photographs, measuring cups, and spoons were utilized. Based on the dietary data, the Korean Healthy Eating Index (KHEI) was calculated [24]. The KHEI comprises eight adequacy components, which assess the intake of recommended foods or dietary habits (e.g., breakfast, mixed grains, total fruits, fresh fruits, total vegetables, vegetables excluding Kimchi and pickled vegetables, meat—fish, eggs, beans, and milk and milk products). It also includes three moderation components, which evaluate the intake of food that should be limited (e.g., sodium, saturated fatty acids, sweets and beverages), and three balance components, which assess energy intake balance (e.g., percent energy from carbohydrates, percent energy from fat, total energy intake). The KHEI scores range from 0 to 100, with higher scores indicating better diet quality.

#### Cardiometabolic health indicators.

Participants’ cardiometabolic health was evaluated using anthropometric measurements and a blood test, stratified by household type. Obesity status was evaluated by calculating body mass index (BMI) from measured height and weight. BMI (kg/m^2^) was calculated by dividing weight (kg) by height squared (m^2^). Abdominal adiposity was assessed by waist circumference, with abdominal adiposity defined based on the Korean Obesity Society’s criteria: ≥ 90 cm for men and ≥85 cm for women [25]. Blood pressure and blood test parameters were measured to identify high blood pressure, dyslipidemia, and impaired fasting glucose. Blood pressure was assessed using both systolic and diastolic values, and blood test parameters included fasting blood glucose, glycated hemoglobin (HbA1c), total cholesterol, triglycerides, HDL-cholesterol level (high density lipoprotein-cholesterol), and LDL-cholesterol level (low density lipoprotein-cholesterol). High blood pressure was defined as systolic blood pressure ≥130 mmHg or diastolic blood pressure ≥85 mmHg. Dyslipidemia was categorized as total cholesterol >200 mg/dL, triglycerides >150 mg/dL, HDL-cholesterol <40 mg/dL for men and <50 mg/dL for women, and LDL-cholesterol >130 mg/dL (Rhee et al., 2019). Impaired fasting glucose was defined as a triglyceride-to-HDL-cholesterol ratio of ≥3, fasting glucose ≥100 mg/dL, or glycated hemoglobin ≥5.7%) [26,27].

#### Quality of life.

Health-related quality of life was assessed using the EuroQol-5 Dimension (EQ-5D), a widely used and validated instrument with demonstrated reliability in various studies [28]. The EQ-5D evaluates five dimensions of health: mobility, self-care, usual activities, pain/discomfort, and anxiety/depression. Each dimension was origin ally rated on three levels: “no problems,” “some problems,” and “extreme problems” [29]. For the purposes of this study, these levels were dichotomized into binary categories, indicating the presence or absence of problems in each dimension. In addition, the EQ-5D index score was calculated following the scoring algorithm provided by the Korea Disease Control and Prevention Agency. If no problems are reported [30].

### Statistical analysis

Data are presented as proportions (standard error, SE) for categorical variables and as the mean with 95% confidence intervals (CIs) for continuous variables. Standard errors were estimated using Taylor series linearization to account for the complex survey design. A pooled weight, calculated based on the number of sample units, was applied to analyze the combined data from the 2013–2021 KNHANES.

All statistical analyses were conducted separately for men and women. Differences in continuous variables by household type were compared using independent t-tests, and categorical variables were compared using the Rao-Scott χ^2^ tests.

Diet quality, assessed using the KHEI, was compared across household types using a multivariate regression model. The analysis included participants who provided information on all covariates (men = 17,215, women = 23,573). Potential confounders adjusted for in the models included age (years), total energy intake (kcal), household income (low, middle-low, middle-high, high), smoking status (never smoked, former smoker, current smoker), alcohol consumption (non-drinker, consumes one or more alcohol drinks per month), and use of dietary supplements (yes/no), and body mass index categories (< 23 kg/m^2^, 23–25 kg/m^2^, ≥ 25 kg/m^2^). For women, menopausal status was additionally adjusted.

Associations between cardiometabolic health indicators and household type were assessed separately for men and women using multivariate logistic regression models. Multivariate logistic regression analyses included participants with complete covariate data (men = 17,215, women = 23,573) and adjusted for the variables described above. To assess whether the association between household type and cardiometabolic outcomes varied by sex, we included an interaction term (sex * household type) in the logistic regression analysis.

Quality of life, assessed using the EQ-5D, was also compared across household types using a multivariate regression model. This analysis included participants who provided complete covariate information (men = 14,456, women = 20,022) and adjusted for the potential confounders described above. To evaluate whether associations differed by sex, an interaction term (sex*household type) was included in the regression model.

Subgroup analyses were conducted to explore the associations between health-related factors and household type by age. Data were analyzed using SAS Software version 9.4 (SAS Institute Inc., Cary, NC, USA), and differences were considered significant at p < 0.05.

## Results

A total of 40,839 participants aged 19–64 years were included in the study (17,234 men and 23,605 women). Table 1 presents the descriptive characteristics of the study population, including demographic information and health-related behaviors. Among participants under 40 years of age, men were nearly twice as likely as women to live in single-person households, whereas among those aged 60 and above, women were 2.8 times more likely than men to reside in single-person households. Regarding educational level, individuals in multi-person households tended to have higher education levels. However, among women in single-person households, the highest proportion had the lowest educational level. Additionally, 50% of the women living in single-person households had low-income status. In terms of marital status, widowhood rates were markedly higher among all women, but particularly for those living in single-person households. Smoking rates were also higher for both men and women in single-person households, with women in this group having nearly double the smoking rate of women in multi-person households. While non-drinking rates were higher among women than men overall, men tended to drink more frequently when living alone, whereas women showed reduced alcohol consumption when living alone. Furthermore, women, regardless of household composition, had higher dietary supplement usage rates than men.

**Table 1:** Basic characteristics of the study population by household type and gender.

	Men (n = 17,234)			Women (n = 23,605)		
	Multi-person household (n = 15,368)	Single-person household (n = 1,866)	p-value	Multi-person household (n = 20,584)	Single-person household (n = 3,021)	p-value
Age			<.0001			<.0001
19–39 yrs	38.4(0.5)	47.8(1.8)		35.9(0.4)	24.6(1.5)	
40–59 yrs	42.3(0.5)	32.3(1.4)		42.8(0.4)	18.7(0.9)	
60 yrs or more	19.3(0.4)	19.9(1.0)		21.4(0.4)	56.6(1.5)	
Education level			0.0208			<.0001
Middle school or below	15.3(0.4)	18.5(1.1)		23.0(0.4)	51.7(1.5)	
High school	39.1(0.5)	37.7(1.5)		36.4(0.5)	21.8(1.1)	
College or above	45.6(0.6)	43.8(1.6)		40.5(0.5)	26.6(1.4)	
Household income			<.0001			<.0001
Low	10.4(0.3)	29.9(1.4)		12.1(0.4)	50.2(1.3)	
Middle-low	23.2(0.5)	21.0(1.1)		24.1(0.4)	25.6(1.0)	
Middle-high	30.8(0.5)	24.5(1.3)		30.1(0.5)	15.6(1.0)	
High	35.6(0.7)	24.6(1.4)		33.7(0.6)	8.6(0.7)	
Marital status			<.0001			<.0001
Married	71.5(0.5)	12.5(0.9)		71.3(0.4)	9.1(0.6)	
Separated or divorced	2.3(0.1)	17.5(1.0)		4.0(0.2)	15.8(0.8)	
Widowed	0.7(0.1)	6.6(0.5)		6.2(0.2)	46.2(1.3)	
Never married	25.5(0.5)	63.4(1.6)		18.5(0.4)	29.0(1.5)	
Smoking status			<.0001			<.0001
Never smoker	30.5(0.4)	28.2(1.4)		90.4(0.3)	83.6(0.9)	
Former smoker	34.5(0.4)	26.5(1.1)		4.5(0.2)	6.6(0.5)	
Current smoker	35.0(0.5)	45.3(1.4)		5.1(0.2)	9.9(0.8)	
Monthly alcohol consumption (1 or more/month)			0.0473			<.0001
No	28.3(0.4)	25.8(1.1)		54.8(0.4)	61.1(1.3)	
Yes	71.7(0.4)	74.2(1.1)		45.2(0.4)	38.9(1.3)	
Dietary supplement use			0.1311			0.0041
No	52.6(0.5)	50.2(1.5)		42.1(0.4)	38.7(1.1)	
Yes	47.4(0.5)	49.8(1.5)		57.9(0.4)	61.3(1.1)	

Values are presented as the mean (standard error, s.e.). p from Rao-Scott chi-square test.

Table 2 compares overall dietary quality, assessed by the KHEI scores, between men and women according to household composition. The KHEI, consisting of 14 components, indicated that women consistently achieved higher scores than men, regardless of the household type. Among men, the KHEI score was approximately five points lower in single-person households compared to multi-person households, reflecting a notable decline in dietary quality. In contrast, for women living in single-person households, the score decreased by less than 1 point, indicating only a marginal difference in overall dietary quality. While men in single-person households exhibited consistently lower overall KHEI scores, women in single-person households demonstrated higher scores in specific aspects, including breakfast consumption, whole grain intake, the percentage of energy from saturated fats, and sodium intake.

**Table 2:** Korean Healthy Eating Index by household type and gender.

	Men (n = 17,215)				Women (n = 23,573)		
	Score range	Multi-person household (n = 15,351)	Single-person household (n = 1,864)		Multi-person household (n = 20,554)	Single-person household (n = 3,019)	
		Mean (95% CI)	Mean (95% CI)	p-value^a^	Mean (95% CI)	Mean (95% CI)	p-value^a^
Total score	0-100	60.54(60.27-60.8)	54.98(54.26-55.71)	<.0001	62.8(62.55-63.05)	61.94(61.24-62.64)	<.0001
Adequacy (8)							
Have breakfast	0-10	6.92(6.84-7.01)	5.26(5.01-5.5)	<.0001	6.98(6.9-7.05)	7.29(7.07-7.51)	<.0001
Mixed grains intake	0-5	1.99(1.94-2.03)	1.2(1.09-1.3)	<.0001	2.01(1.98-2.05)	2.04(1.93-2.14)	0.0003
Total fruits intake	0-5	1.72(1.68-1.76)	1.17(1.07-1.27)	<.0001	2.5(2.46-2.55)	2.36(2.26-2.47)	<.0001
Fresh fruits intake	0-5	1.92(1.88-1.97)	1.27(1.15-1.38)	<.0001	2.7(2.66-2.74)	2.46(2.34-2.57)	<.0001
Total vegetables intake	0-5	3.76(3.73-3.79)	3.51(3.42-3.6)	0.0016	3.21(3.18-3.23)	3.16(3.08-3.23)	<.0001
Vegetables intake^*^	0-5	3.38(3.35-3.41)	3.08(2.99-3.17)	0.0006	3.05(3.02-3.08)	3.05(2.97-3.13)	0.0142
Meat, fish, eggs, and beans intake	0-10	7.52(7.46-7.58)	7.31(7.14-7.48)	0.9294	6.93(6.88-6.99)	6.56(6.41-6.71)	0.3351
Milk and milk products intake	0-10	3.03(2.94-3.11)	2.84(2.61-3.08)	0.6610	3.63(3.55-3.71)	3.51(3.3-3.72)	<.0001
Moderation (3)							
Percentage of energy from saturated fatty acids	0-10	7.21(7.13-7.29)	6.67(6.42-6.92)	<.0001	7.27(7.2-7.34)	7.66(7.46-7.86)	<.0001
Sodium intake	0-10	5.44(5.37-5.51)	5.66(5.47-5.85)	0.5503	7.55(7.5-7.6)	8.07(7.96-8.18)	<.0001
Percentage of energy from sweets and beverages	0-10	8.41(8.35-8.47)	8.13(7.94-8.31)	0.0040	8(7.94-8.07)	7.84(7.67-8.02)	0.0004
Energy balance (3)							
Percentage of energy from carbohydrates	0-5	2.68(2.64-2.72)	2.56(2.44-2.67)	0.3701	2.53(2.5-2.57)	2.09(2-2.18)	0.4654
Percentage of energy intake from fat	0-5	3.47(3.43-3.51)	3.34(3.23-3.45)	0.3636	3.38(3.35-3.41)	2.89(2.8-2.98)	0.0101
Energy intake	0-5	3.09(3.05-3.13)	3.01(2.88-3.13)	0.5886	3.05(3.01-3.08)	2.97(2.87-3.06)	0.4469

* Excluding Kimchi and pickled vegetables intake.

^a^Comparing the Korean Healthy Eating Index score and its components by household type adjusting for age, total energy intake, household income, smoking status, alcohol consumption, body mass index, and dietary supplements use.

Table 3 compares individuals in single-person households with those in multi-person households and presents the ORs and 95% CIs for cardiometabolic health indicators. Among men, living alone was associated with a significantly higher risk of several adverse health indicators, including elevated waist circumference, LDL-cholesterol, total cholesterol, and blood pressure. Conversely, among women, residing in single-person households was associated with a 10–20% reduction in the risk of elevated BMI, triglycerides, LDL-cholesterol, total cholesterol, and fasting blood glucose. Notably, significant sex differences were confirmed by interaction terms for BMI (p = 0.011), LDL-cholesterol (p = 0.002), total cholesterol (p < 0.001), fasting blood glucose (p = 0.004), and elevated blood pressure (p = 0.036), indicating that association between household type and the these cardiometabolic indicators varies significantly by sex.

**Table 3:** Odds ratios (ORs) and 95% confidence intervals (CIs) for cardiometabolic health indicators based on single-person household status.

	Single-Person Household		p for interaction
Variable	Men	Women	
Body mass index (≥25 kg/m^2^)	0.90(0.80-1.02)	0.82(0.74-0.91)	0.0109
Waist circumference^a^	1.21(1.02-1.43)	1.06(0.91-1.24)	0.7974
Triglycerides (≥150 mg/dl)	0.98(0.86-1.11)	0.84(0.75-0.95)	0.6293
High-density lipoprotein cholesterol (<40 mg/dl)	0.99(0.86-1.13)	0.96(0.83-1.10)	0.4845
Low-density lipoprotein cholesterol (≥130 mg/dl)	1.14(1.00-1.30)	0.81(0.73-0.90)	0.0017
Total cholesterol (≥200 mg/dl)	1.13(1.01-1.28)	0.80(0.72-0.89)	0.0003
HbA1c (≥5.7%)	0.95(0.83-1.09)	0.96(0.86-1.07)	0.7457
Fasting blood glucose (≥100 mg/dl)	1.13(0.99-1.28)	0.89(0.80-0.99)	0.0039
Blood pressure (130 ≤ systolic blood pressure or 85 ≤ diastolic blood pressure)	1.35(1.19-1.54)	0.96(0.85-1.08)	0.0358

^a^Over 90 cm for men, over 85 cm for women. Reference group is multi-person household. Multivariate logistic regression analyses were conducted, adjusting for age, total energy intake, household income, smoking status, alcohol consumption, body mass index, and dietary supplement use.

Table 4 displays the odds ratios for cardiometabolic risk factors in single-person households compared to multi-person households, stratified by gender and age group. Among men aged 40−59 years, living in single-person households was associated with significantly lower odds of having a BMI ≥ 25 kg/m² compared to those in multi-person households (OR: 0.81, 95% CI: 0.65–1.00). For women, younger individuals (19−39 years) in single-person households exhibited significantly lower odds of greater waist circumference (OR: 0.63, 95% CI: 0.40–0.98), while women aged 40−59 years exhibited lower odds of BMI (OR: 0.68, 95% CI: 0.54–0.85). Women over 60 years demonstrated significantly lower odds of elevated triglycerides (OR: 0.87, 95% CI: 0.76–0.99) but higher odds of elevated HbA1c levels (OR: 1.19, 95% CI: 1.04–1.36). In contrast, younger men (19−39 years) living in single-person households had significantly higher odds of elevated LDL-cholesterol (OR: 1.32, 95% CI: 1.07–1.64) and total cholesterol (OR: 1.26, 95% CI: 1.02–1.55). Additionally, both younger men and middle-aged men (40−59 years) demonstrated higher odds of elevated blood pressure compared to their counterparts in multi-person households (OR: 1.47, 95% CI: 1.17–1.86 for younger men; OR: 1.48, 95% CI: 1.20–1.82 for middle-aged men). Middle-aged men in single-person households also exhibited significantly higher odds of elevated fasting blood glucose (OR: 1.33, 95% CI: 1.08–1.62) compared to their peers in multi-person households.

**Table 4:** Odds ratios and 95% confidence intervals for cardiometabolic health indicators by gender and age group.

	Single-Person Household					
Variable	Men			Women		
	19 ~ 39 y	40 ~ 59 y	Over 60 y	19 ~ 39 y	40 ~ 59 y	Over 60 y
Body mass index (≥25 kg/m^2^)	0.95 (0.78-1.16)	0.81 (0.65-1.00)	0.99 (0.82-1.21)	0.94 (0.69-1.28)	0.68 (0.54-0.85)	0.97 (0.86-1.10)
Waist circumference^a^	1.18 (0.86-1.60)	1.27 (0.95-1.70)	1.20 (0.93-1.55)	0.63 (0.40-0.98)	1.08 (0.77-1.51)	1.06 (0.90-1.27)
Triglycerides (≥150 mg/dl)	0.83 (0.67-1.03)	1.23 (0.99-1.53)	1.00 (0.82-1.21)	1.02 (0.70-1.48)	1.11 (0.86-1.44)	0.87 (0.76-1.00)
High-density lipoprotein cholesterol (<40 mg/dl)	1.00 (0.78-1.28)	1.00 (0.80-1.25)	0.93 (0.76-1.15)	0.62 (0.32-1.20)	1.05 (0.74-1.49)	0.98 (0.83-1.17)
Low-density lipoprotein cholesterol (≥130 mg/dl)	1.32 (1.07-1.64)	0.98 (0.79-1.23)	1.06 (0.86-1.32)	0.97 (0.71-1.33)	1.03 (0.83-1.27)	0.93 (0.82-1.07)
Total cholesterol (≥200 mg/dl)	1.26 (1.02-1.55)	1.09 (0.88-1.34)	1.00 (0.82-1.23)	0.93 (0.70-1.22)	1.17 (0.95-1.44)	0.90 (0.79-1.03)
HbA1c (≥5.7%)	1.02 (0.79-1.31)	1.00 (0.81-1.24)	0.84 (0.69-1.02)	0.92 (0.63-1.33)	0.89 (0.72-1.09)	1.19 (1.04-1.36)
Fasting blood glucose (≥100 mg/dl)	1.12 (0.86-1.44)	1.33 (1.08-1.62)	1.02 (0.84-1.23)	0.95 (0.65-1.40)	0.97 (0.77-1.21)	0.98 (0.87-1.11)
Blood pressure (130 ≤ systolic blood pressure or 85 ≤ diastolic blood pressure)	1.47 (1.17-1.86)	1.48 (1.20-1.82)	1.16 (0.96-1.39)	1.25 (0.79-1.99)	1.25 (0.99-1.58)	0.94 (0.82-1.07)

^a^Over 90 cm for men, over 85 cm for women. Reference group is multi-person household. Multivariate logistic regression analyses were conducted, adjusting for age, total energy intake, household income, smoking status, alcohol consumption, body mass index, and dietary supplement us.

Analysis of the quality of life index revealed significant differences between single-person and multi-person households for both genders (Table 5). Among men, individuals in multi-person households had significantly higher quality of life scores (0.970, 95% CI: 0.969–0.972) compared to those in single-person households (0.953, 95% CI: 0.948–0.959, p = 0.0015). The disparity was more pronounced for women, with multi-person households scoring higher (0.953, 95% CI: 0.951–0.954) than single-person households (0.899, 95% CI: 0.892–0.906, p < 0.0001, p for interaction by sex = 0.0046). The Rao-Scott chi-square test revealed significant differences across all EQ-5D dimensions between single-person and multi-person households for both genders (Table 6). Among women, those in single-person households reported more problems, particularly in mobility (26.97% vs. 10.47%), usual activities (15.32% vs. 5.83%), and pain/discomfort (35.76% vs. 23.32%). Anxiety/depression was also more prevalent for women living in single-person households (17.40% vs 10.62%). Similarly, men living in single-person households experienced more problems with anxiety/depression (11.29% vs. 5.72%), mobility (10.53% vs. 6.76%), and pain/discomfort (19.68% vs 14.98%), although the differences were less pronounced compared to women.

**Table 5:** Quality of life index (EQ-5D index score) by household type and gender.

	N	Mean (95% CI)	p-value	P for interaction
Men			0.0015	0.0046
Multi-person household	12,951	0.97(0.97-0.97)		
Single-person household	1,505	0.95(0.95-0.96)		
Women			<.0001	
Multi-person household	17,630	0.95(0.95-0.95)		
Single-person household	2,392	0.90(0.89-0.91)		

If no problems are reported across all five dimensions, the EQ-5D index score is 1. Comparing the EQ-5D index score by household type adjusting for age, total energy intake, household income, smoking status, alcohol consumption, body mass index, and dietary supplement us.

**Table 6:** Item-specific differences in the quality of life (EQ-5D) index by household type and gender.

	Men			Women		
	Multi-person household weighted %	Single-person household weighted %	p-value	Multi-person household weighted %	Single-person household weighted %	p-value
Mobility			<.0001			<.0001
no problems	93.24	89.47		89.53	73.03	
problems	6.76	10.53		10.47	26.97	
Self-Care			<.0001			<.0001
no problems	98.39	96.97		97.71	93.06	
problems	1.61	3.03		2.29	6.94	
Usual Activities			<.0001			<.0001
no problems	96.36	93.03		94.17	84.68	
problems	3.64	6.97		5.83	15.32	
Pain/Discomfort			<.0001			<.0001
no problems	85.02	80.32		76.68	64.24	
problems	14.98	19.68		23.32	35.76	
Anxiety/Depression			<.0001			<.0001
no problems	94.28	88.71		89.38	82.60	
problems	5.72	11.29		10.62	17.40	

The term “problem” includes both “some problems” and “extreme problems.” p from Rao-Scott chi-square test.

Subgroup analyses of the EQ-5D index by age and gender (Table 7) showed significant differences between middle-aged men (40−59 years) and older men (≥60 years) living in single-person households. Middle-aged men in single-person households also had lower quality of life scores (0.946, 95% CI: 0.933–0.959) compared to those in multi-person households (0.975, 95% CI: 0.973–0.977, p = 0.004). Similarly, older men in single-person households scored lower (0.906, 95% CI: 0.895–0.918) than their multi-person counterparts (0.940, 95% CI: 0.936–0.944, p = 0.013). However, no statistically significant differences were observed for women across age groups, although the trends generally indicated lower scores for single-person households in older age groups.

**Table 7:** Subgroup analysis of quality of life (EQ-5D) index by household type stratified by gender and age.

Age (years)	N	Mean (95% CI)	p-value
**Men**			
19-39			0.873
Multi-person household	3,857	0.980(0.978-0.982)	
Single-person household	494	0.977(0.978-0.982)	
40-59			0.004
Multi-person household	4,875	0.975(0.973-0.977)	
Single-person household	427	0.946(0.933-0.959)	
≥60			0.013
Multi-person household	4,219	0.940(0.936-0.944)	
Single-person household	584	0.906(0.895-0.918)	
**Women**			
19-39			0.447
Multi-person household	5,300	0.973(0.971-0.974)	
Single-person household	299	0.971(0.966-0.977)	
40-59			0.071
Multi-person household	7,527	0.963(0.961-0.965)	
Single-person household	451	0.937(0.926-0.948)	
≥60			0.213
Multi-person household	4,803	0.896(0.891-0.901)	
Single-person household	1,642	0.852(0.842-0.861)	

Comparing the EQ-5D index score by household type adjusting for age, total energy intake, household income, smoking status, alcohol consumption, body mass index, and dietary supplement use.

## Discussion

This study analyzed differences in dietary habits, cardiometabolic health indicators, and health-related quality of life between single-person and multi-person households in Korea, using data from the 2013–2021 Korea National Health and Nutrition Examination Survey (KNHANES). While previous research has largely focused on household type itself, concluding that single-person households generally have poorer health, dietary behaviors, and quality of life compared to multi-person households, the increasing diversity among single-person households necessitates a more detailed classification. Such segmentation is essential for developing tailored policies and market strategies targeting the diverse forms of single-person households. Indeed, recent studies have actively classified subgroups within the same age cohort based on characteristics such as income and consumption patterns [31,32]. By segmenting single-person households further by gender and age, this study provided more nuanced insights into the multidimensional disparities associated with household structure.

Analysis of dietary habits showed that single-person male households had consistently lower Korean Healthy Eating Index (KHEI) scores compared to multi-person male households, in line with previous findings indicating higher dependence on dining out and convenience foods among this group. Conversely, single-person female households achieved higher scores in certain components such as breakfast consumption, whole grain intake, and sodium moderation, with little difference observed in overall KHEI scores compared to multi-person female households. This gender difference can be explained by socialized role learning regarding cooking responsibilities within households. Children generally learn dietary and lifestyle behaviors from their parents [33,34]. In Korean households, women are more likely to be responsible for domestic tasks, including meal preparation [35]. Therefore, for single-person male households, acquiring and managing cooking skills independently can become a significant burden. This culturally embedded role learning may partly explain why single-person men rely more on eating out or convenience foods compared to single-person women or multi-person households.

Regarding cardiometabolic health indicators, single-person male households exhibited higher levels of waist circumference, LDL cholesterol, total cholesterol, and blood pressure than multi-person male households. In contrast, single-person female households showed significantly lower levels of BMI, triglycerides, LDL cholesterol, and fasting glucose compared to multi-person female households. This finding contrasts with previous studies reporting that single-person households generally have higher risks of metabolic disorders and health issues compared to multi-person households [36,37]. To further explore these differences, this study stratified single-person households by gender and age. Among single-person women, those aged 19–39 years had a significantly lower likelihood of having increased waist circumference (OR: 0.63, 95% CI: 0.40–0.98) compared to their multi-person counterparts. Similarly, women aged 40–49 years in single-person households had a lower likelihood of high BMI (OR: 0.68, 95% CI: 0.54–0.85), and single women aged 60 years or older showed a reduced likelihood of elevated triglycerides (OR: 0.87, 95% CI: 0.76–0.99) compared to multi-person women. In contrast, all cardiometabolic indicators among single-person men were worse compared to multi-person men. Thus, targeted policy programs aimed at improving cardiometabolic health among single-person male households are urgently needed. Meanwhile, since some age groups of single-person female households showed better metabolic profiles, a more segmented approach is needed when analyzing the health status of single-person women. One possible interpretation is that lower BMI and waist circumference values observed among women in single-person households may reflect internalized sociocultural beauty norms rather than better physical health per se. This remains a hypothesis, and further research is needed to examine the extent to which body image perceptions and appearance-related motivations influence dietary and lifestyle behaviors in this group.

Although the cardiometabolic profiles of single-person women appeared better than those of multi-person women, careful interpretation is necessary. Previous studies have suggested that women internalize sociocultural beauty norms, promoting behaviors such as weight management [38,39]. BMI and waist circumference are often perceived as indicators of socially desirable physical appearance [40]. Thus, the better BMI and waist circumference observed among single-person women in this study may be partially attributable to the internalization of such beauty standards rather than healthier lifestyle factors. Further research is required to clarify the influence of sociocultural body image norms on these findings.

In terms of quality of life, single-person female households reported more problems related to mobility, usual activities, pain/discomfort, and anxiety/depression compared to multi-person female households. This suggests that although single-person women may have better physical health indicators, they may still experience greater psychological vulnerabilities and lower overall life satisfaction. Middle-aged and older men in single-person households also demonstrated significantly lower quality of life than their multi-person counterparts, suggesting the need for greater attention to this group’s overall well-being.

**Fig 1:**
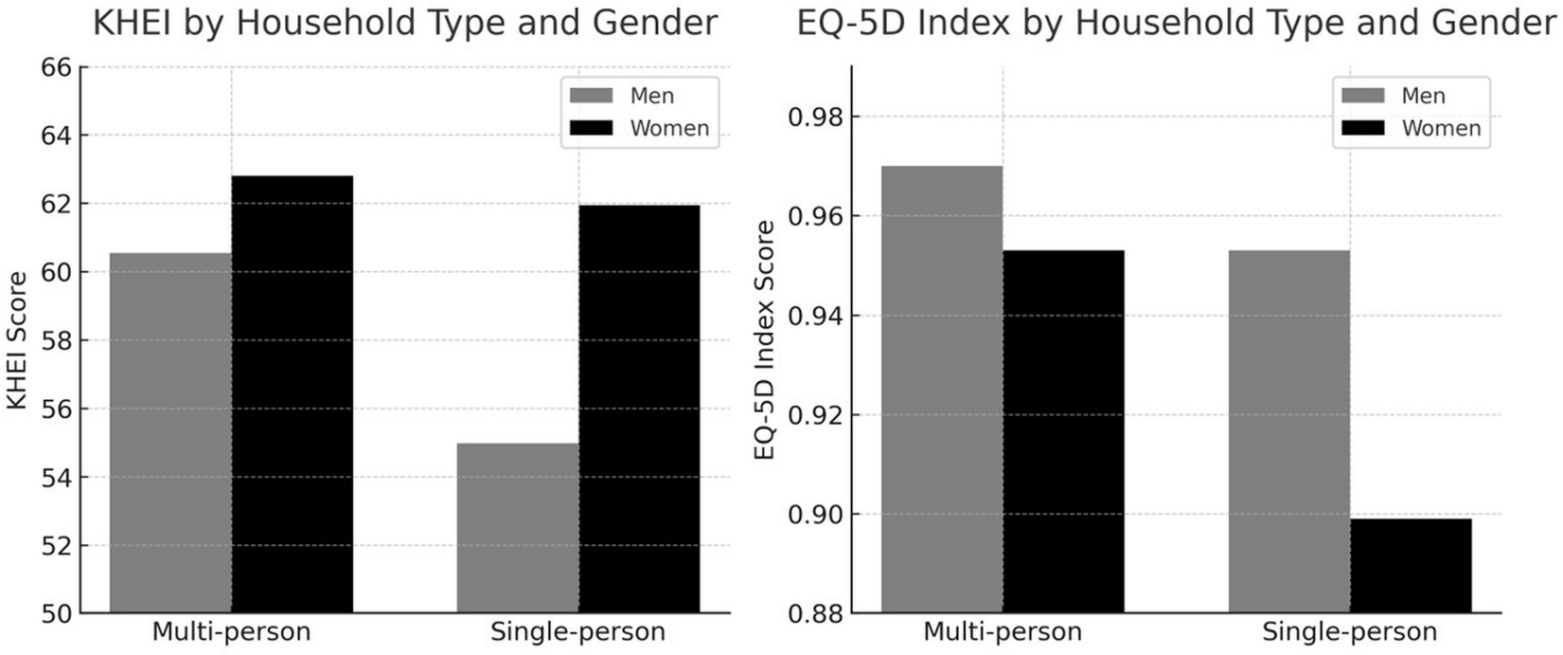
Comparison of dietary quality and health-related quality of life by household type and gender. The figure presents mean scores for the Korean Healthy Eating Index (KHEI) and the EQ-5D index, stratified by household type (single-person vs. multi-person) and gender. Single-person male households showed the lowest dietary quality, while single-person female households reported the lowest quality of life scores.

Beyond these differences in life quality, single-person male households further exhibited the lowest dietary quality, as reflected by their scores on the KHEI. The combined differences in dietary behavior and health-related quality of life are visually summarized in Fig 1, which compares KHEI and EQ-5D scores by household type and gender.

Thus, both single-person female households and middle-aged and older single-person male households should be prioritized for policy interventions targeting not only physical health improvement but also psychological and social support. Given the steady increase in single-person households globally, improving their quality of life is an urgent policy task. Moreover, from a market perspective, there is a need to develop new services such as mental health counseling programs and community engagement programs aimed at addressing the specific needs of single-person households.

This study offers important implications for public health and market strategies. Tailored health promotion programs aimed at improving dietary habits and preventing metabolic disorders should be developed for single-person male households. Psychological support services should be strengthened for single-person female households. Considering the growing reliance on convenience foods among single-person households, private sectors should also expand efforts to develop nutritionally balanced meal solutions tailored to this population. Single-person households are no longer a homogeneous group; gender- and age-specific policy designs are crucial to addressing health inequalities and promoting social integration.

Nevertheless, this study has some limitations. First, due to its cross-sectional design, causal relationships cannot be inferred. Second, the data were based on self-reported information, making them susceptible to recall bias and reporting errors. Third, unmeasured confounding variables, such as mental health service utilization, may have influenced the findings. Lastly, as this study was conducted within the Korean context, its findings may not be fully generalizable to other cultural settings. Future studies should segment single-person households in various countries and cultural contexts to identify differing patterns of vulnerability, health disparities, and policy needs. Longitudinal research, qualitative studies using in-depth interviews, and international comparative studies are needed to build a more systematic understanding of the health behaviors and quality of life differences among single-person households.

## Conclusions

This study compared health, dietary habits, and quality of life (QoL) single-person and multi-person households using data from the Korean National Health and Nutrition Examination Survey (KNHANES). The results showed significant differences, especially highlighting vulnerabilities of specific subgroups such as single-male households and elderly single-female households. Single-person male households had lower Korean Healthy Eating Index (KHEI) scores and lower metabolic health indicators such as higher waist circumference, LDL cholesterol, and blood pressure compared to multi-person households. In contrast, single-female households had slightly better metabolic profiles, with lower BMI and fasting glucose levels, but significantly lower QoL, particularly in terms of mobility, pain/discomfort, and anxiety/depression. The EQ-5D scores further reflected lower quality of life among single-person households, with middle-age and older single-male households and single-family households showing particularly significant differences.

These findings highlight the need for tailored public health policies that consider the gender and age specific characteristics of single-person households. Interventions should focus on improving diet quality and addressing cardiometabolic risk. They should also provide mental health support for single-person households. Furthermore, the increasing reliance on convenience foods in single-person households presents an opportunity to promote nutritionally balanced options tailored to their needs. Future research should also focus on examining single-person households in different cultural contexts and explore long-term trends to develop more targeted strategies. Such efforts are essential to address health disparities and improve the quality of life of this growing population.

## Abbreviations

BMI: body mass index
CIs: confidence intervals
EQ-5D: EuroQol-5 Dimension
HDL-cholesterol: High-density lipoprotein cholesterol
KHEI: Korean Healthy Eating Index
KNHANES: Korean National Health and Nutrition Examination Survey
LDL-cholesterol: Low-density lipoprotein cholesterol
ORs: odds ratios
SE: standard error
